# Sexual behavior and testis morphology in the BACHD rat model

**DOI:** 10.1371/journal.pone.0198338

**Published:** 2018-06-08

**Authors:** Arianna Novati, Libo Yu-Taeger, Irene Gonzalez Menendez, Leticia Quintanilla Martinez, Huu Phuc Nguyen

**Affiliations:** 1 Institute of Medical Genetics and Applied Genomics, University of Tübingen, Tübingen, Germany; 2 Centre for Rare Diseases, University of Tübingen, Tübingen, Germany; 3 Institute of Pathology and Neuropathology and Comprehensive Cancer Center, University of Tübingen, Tübingen, Germany; 4 Department of Human Genetics, University of Bochum, Bochum, Germany; Emory University, UNITED STATES

## Abstract

**Background:**

Huntington disease (HD) is an autosomal dominant neurodegenerative disorder caused by a mutation in the huntingtin (HTT) gene, which results in brain neurodegeneration and peripheral pathology affecting different organs including testis. Patients with HD suffer from motor and cognitive impairment, and multiple psychiatric symptoms. Among behavioral abnormalities in HD, sexual disturbances have often been reported, but scarcely investigated in animal models. The BACHD rat model of HD carries the human full-length mutated HTT (mHTT) genomic sequence with 97 CAG-CAA repeats and displays HD-like alterations at neuropathological and behavioral level.

**Objective:**

This study aims to phenotype the BACHD rats’ sexual behavior and performance as well as testis morphology because alterations in these aspects have been associated to HD.

**Methods:**

Two rat cohorts at the age of 3 and 7 months were subjected to mating tests to assess different parameters of sexual behavior. Histological analyses for testis morphology were performed in different rat cohorts at 1.5, 7 and 12 months of age whereas immunohistochemical analyses were carried out at 7 and 12 months of age to visualize the presence of mHTT in testicular tissue. Furthermore, western blot analyses were used to assess HTT and mHTT expression levels in striatum and testis at three months of age.

**Results:**

At 3 months, BACHD rats showed a decreased time exploring the female anogenital area (AGA), decreased latency to mount, increased number of intromissions and ejaculations and enhanced hit rate. At 7 months, all sexual parameters were comparable between genotypes with the exception that BACHD rats explored the AGA less than wild type rats. Testis analyses did not reveal any morphological alteration at any of the examined ages, but showed presence of mHTT limited to Sertoli cells in transgenic rats at both 7 and 12 months. BACHD rat HTT and mHTT expression levels in testis were lower than striatum at 3 months of age.

**Conclusions:**

The testis phenotype in the BACHD rat model does not mimic the changes observed in human HD testis. The altered sexual behavior in BACHD rats at three months of age could be to a certain extent representative of and share common underlying pathways with some of the sexual disturbances in HD patients. Further investigating the biological causes of the sexual phenotype in BACHD rats may therefore contribute to clarifying the mechanisms at the base of sexual behavior changes in HD.

## Introduction

Huntington disease (HD) is an inherited neurodegenerative disorder caused by a polyglutamine repeated expansion in the huntingtin (HTT) gene [1]. Mutant HTT (mHTT) is expressed in most tissues examined in humans [2] and results in both widespread neurodegeneration throughout the brain and peripheral abnormalities [2,3]. Patients with HD complain of motor impairment, cognitive deficits and multiple psychiatric disturbances [4–6]. Sexual behavior abnormalities have often been described in HD patients, among behavioral symptoms [7–15]. The typology of sexual disorders in HD is diverse with evidence for both hypersexuality [8,12,13,15] and hyposexuality [9,10] as well as changes in sexual interest and paraphilia [9]. While a high percentage of patients suffer of sexual disturbances [9], the underlying mechanisms are not known, nor it is clear to which extend these symptoms are specific for the disease or secondary to other symptoms. More recent research also reported impaired sexual performance and problems with erections in HD [11]. Thus, HD patients suffer a wide variety of sexual dysfunctions of which the biological causes remain unclear.

HD has also been associated with physiological and morphological changes that could be directly or indirectly related to sexual dysfunction. There are alterations of the hypothalamus—pituitary—gonadal activity [16] and testis pathology which consists of changes in seminiferous tubule morphology as well as decreased number of developing germ cells [17]. Mouse and transgenic HD (TgHD) minipig boar models of HD mimic, to a certain extent, the testicular pathology in humans [17,18] and additionally show testis atrophy and degeneration [17–19]. Whereas testicular pathology in YAC128 mice and TgHD minipig boar models was proposed as a local effect of mutant HTT [17,18], testicular alterations in R6/2 mice were suggested to derive from a decreased number of neurons secreting gonadotropin—releasing—hormone in the brain [19]. This indicates that the changes triggering testis pathology in HD could be diverse.

BACHD rats are an established model of HD carrying a construct which contains the full-length human HTT genomic sequence with 97 CAG/CAA repeats and all regulatory elements [20]. These rats show neuropathological, metabolic and behavioral HD related alterations [14,20–27]. The BACHD rat behavioral phenotype includes motor impairment [14,20,21], cognitive deficits [14,24,27] and emotional changes [14,20,21,25,26]. In this study, we extend the characterization of BACHD rats to new phenotypes related to sexual behavior and functionality as well as to testicular morphology, which have been little investigated in HD animal models.

## Material and methods

### Animals and housing

Experiments were performed in BACHD male rats and wild type (WT) littermates. Details on the BACHD rat generation and construct were published previously [20]. Male experimental rats in our study were bred on a Sprague Dawley background by pairing heterozygous BACHD males of the TG5 line [20], born at our facility, with WT females (Charles River, Germany). At postnatal day 21, rats were weaned, ear marked and genotyped following previous protocols [20]. After weaning, experimental male rats were housed in social groups of 4 with same gender littermates of mixed genotype and kept in type IV cages enriched with bedding, nesting material and a wooden house. Ovariectomized Sprague Dawley WT females were purchased from Charles River (Germany) and housed with the same cage conditions as males. Females were 8 weeks old when delivered and were left undisturbed for 3 weeks before starting the mating tests. All experimental animals were kept in a room with constant temperature (22 ± 1 °C) and humidity (55% ± 10%), under 12:12 hours light/ dark cycle (lights on at 4 am, lights off at 16.00 pm).

The experiments were approved by the local ethics committee (Regierungspraesidium Tuebingen). All procedures were performed according to the German Animal Welfare Act and the guidelines of the Federation of European Laboratory Animal Science Associations, based on European Union legislation (Directive 2010/63/EU).

### Mating test

A total of 65 male rats were part of the behavioral experiments. Two cohorts of animals were tested at the age of 3 and 7 months, respectively. The first cohort included 16 WT and 16 BACHD rats while in the second cohort there were 16 WT and 17 BACHD rats.

In order to score the test parameters in blind conditions, all animals were coded in advance. Behavioral experiments were conducted in the dark phase under red light, starting one hour after the beginning of the light phase. Tests were performed in a Plexiglas box (65X45X45 cm) with bedding material. All animals were habituated to the testing box for 30 minutes, 8 days preceding the experiments and later re-acclimatized to the room environment for 1 h before being tested on each session. Testing protocols were adapted from earlier studies [28,29]. In brief, each male rat was exposed to a stimulus female on four test series, applied at eight days distance in order to be able to observe all parameters of interest. The box was cleaned and bedding changed between animals tested on the same day. Rats housed together were not tested on the same day to prevent that sequentially removing rats from the same cage, could affect their behavior during the test. Different females interacted with the same male on different test series and every female served as stimulus only once on each testing day. All stimulus females received estradiol benzoate (10 μg/0.1 ml in sesame oil; Caelo, Germany) and progesterone (500 μg/0.1 ml in sesame oil; Caelo, Germany), 48 and 4 hours before the test respectively to induce receptivity. Before each mating test, each stimulus female was paired to a non—experimental male to prove the presence of lordosis.

The number of mounts (no vaginal penetration), intromission (vaginal penetration), ejaculations as well as latency to mount and post ejaculatory refractory period were scored during thirty minutes male female interaction on each test session. The hit rate was then calculated as the ratio between the total number of intromissions (I), including ejaculations, divided by the sum of the number of mounts (M) and total number of intromissions (I): (I/M+I) and was used as measure of copulatory efficiency [30]. The post ejaculatory refractory period was calculated as the time between the first ejaculation and the following mount. The time spent sniffing the anogenital area (AGA) was scored in the first five minutes of test.

### Testis analyses

#### Histology and immunohistochemistry

Testis analyses were performed in different animal cohorts at 1.5, 7 and 12 months of age (N = 3-5/group). Testicles were removed from rat bodies directly after sacrifice and immediately fixed in 4,5% formalin. Following 24h, testicles were cut into halves, and 24h later, the resulting testicle pieces were further cut in halves. Fixed testis tissue was embedded in paraffin and cut in sections of 3–5 μm thickness. Paraffin sections from all age cohorts were stained with hematoxylin and eosin (H&E) and used for morphometric analyses and germinal epithelium cell counts.

The number of Sertoli cells and cells belonging to different maturation stages through the germinal epithelium (spermatogonia, primary spermatocytes, spermatids, spermatozoa) was manually counted in 3 animals per group in H&E sections. For each animal, 4 randomly selected tubules were analyzed. Cells of different type were distinguished by morphology and position within the tubules.

The cross-section area of the seminiferous tubules and the area of the germinal epithelium were measured in 3–5 animals per group in H&E sections as previously described [31]. For each animal 90–100 tubules were randomly selected for analysis. Tubules were then classified as reduced (19x10^3^–51x10^3^ μm^2^), small (52x103–83x10^3^ μm^2^), medium (84x10^3^–115x10^3^ μm^2^) and large (116x10^3^–147x10^3^ μm^2^) based on their area [31] and the percentage of tubules belonging to each size group was calculated. Additionally, the presence of degenerative alterations including changes in the normal position of cell types in different maturation stages, presence of cytoplasmic vacuoles in the tubular epithelium, multinucleated spermatids, necrotic Leydig cells, desquamation of the germinal epithelium, congestion of blood vessels and deformation of the interstitial tissue were investigated.

Moreover, the presence of mHTT protein was examined using immunohistochemistry. For this purpose, paraffin sections obtained from testis of 7 and 12 months old animals (N = 4/group/age), were immunostained with anti-HTT protein EM48 antibody (1: 50; MAB5374, Merk—Millipore) and secondary rabbit anti-mouse antibody (1:500, ab133469, Abcam) on an automated Immunostainer (Ventana Medical Systems, Inc.) according to the company’s protocols for open procedures with slight modifications. Appropriate positive and negative controls were used to confirm the specificity of the staining.

#### Western blot analyses

Western blot analyses were performed to assess WT HTT and mHTT protein expression levels in striatum and testis of male BACHD rats and WT littermates (N = 3) at 3 months of age. Testicular and striatal tissues were homogenized in modified RIPA buffer (150 mM sodium chloride, 1.0% NP-40, 0.5% sodium deoxycholate, 0.1% SDS, 50 mM Tris, 5 mM EDTA pH8.0). The same amount of protein of each sample was separated using 7% Tris-Acetate Gel (EA03585BOX of ThermoFische Scientific), and blots were probed with monoclonal antibodies anti-huntingtin protein MAB2166 (1:1000, mouse, Merk—Millipore) and anti-huntingtin D7F7 (1:1000, rabbit, Cell Signaling), recognizing the amino acid 181–810 and residues surrounding Pro1220 of human huntingtin, respectively. Since actin, GAPDH and beta-tubulin expression levels differ between tissues [17], we used Ponceau red staining to confirm equal loading of proteins.

### Statistics

Statistical analyses were performed with Graph Pad Prism 7. Behavioral test parameters measured over testing series were analyzed with Repeated Measure (RM) ANOVA followed by Tukey or Sidak post hoc test when appropriated. The entire sample size could not be used to calculate all behavioral parameters. The number of animals included in the analysis of each parameter is reported in the figure legends. Animals that did not display mounts and intromissions in one or more of the testing series had to be excluded from the hit rate RM ANOVA analyses. The post ejaculatory refractory period in each series could not be estimated for animals that did not perform any ejaculation or ejaculated too late in a testing session. Because this happened frequently in the first two series, RM ANOVA for post ejaculatory period could not be applied through all testing series and genotypes were statistically compared only for the fourth test series, when performance was the highest, using Mann—Whitney test at 3 months and t-test at 7 months.

Germinal epithelium area and tubular area were compared between genotypes using t-test for each age. The percentage of tubules belonging to each size group and the number of cells counted through different stages of the germinal epithelium were analyzed with two-way ANOVA for each age cohort. Significance level was set at p < 0,05 for all statistical tests applied.

## Results

### Mating test

**Fig 1:**
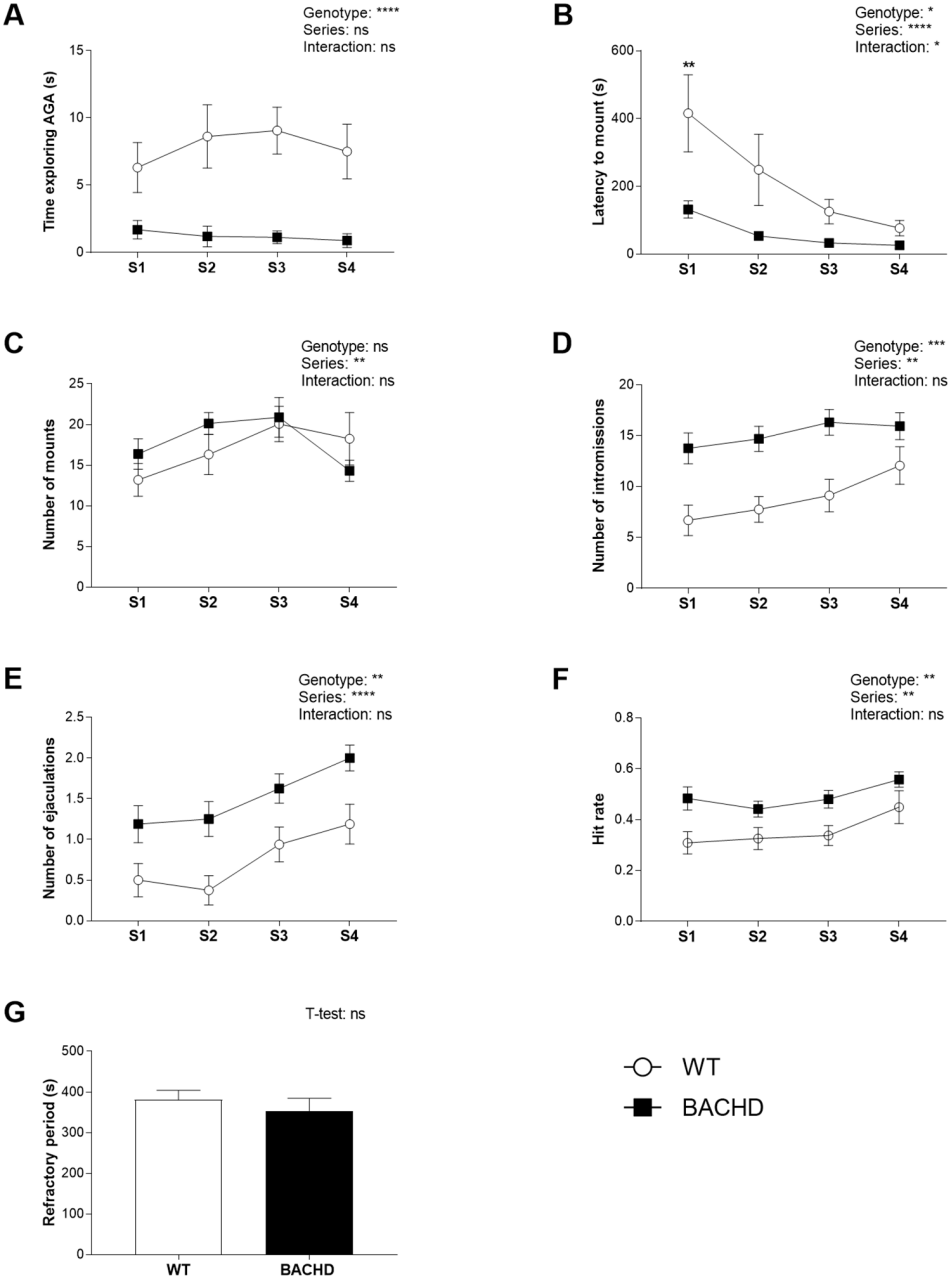
Sexual behavior at 3 months of age. BACHD rats displayed shorter AGA exploration (A) and latency to mount the female (B) compared to WT rats and showed increased number of intromissions (D) and ejaculations (E) and enhanced hit rate (F). The number of mounts (C) and the post ejaculatory refractory period (G) were not significantly different between genotypes. Values in the graphs indicate group mean and standard error for different test series. T-test and repeated measures ANOVA results are reported above the graphs. Results from post-hoc analysis are shown in the graphs for series in which genotypes differed significantly. Series differences are not displayed on graphs. * (p < 0.05), ** (p < 0.01), *** (p < 0.001), **** (p < 0.0001), ns (not significant). N differed among parameters as explained in section 2.4. For the number of mounts, intromission and ejaculations as well as latency to mount N = 16 WT and 16 BACHD. For post ejaculatory refractory period N = 10 WT and 16 BACHD. For hit rate, N = 15 WT and 16 BACHD. AGA = anogenital area; S = series; WT = wild type.

**Fig 2:**
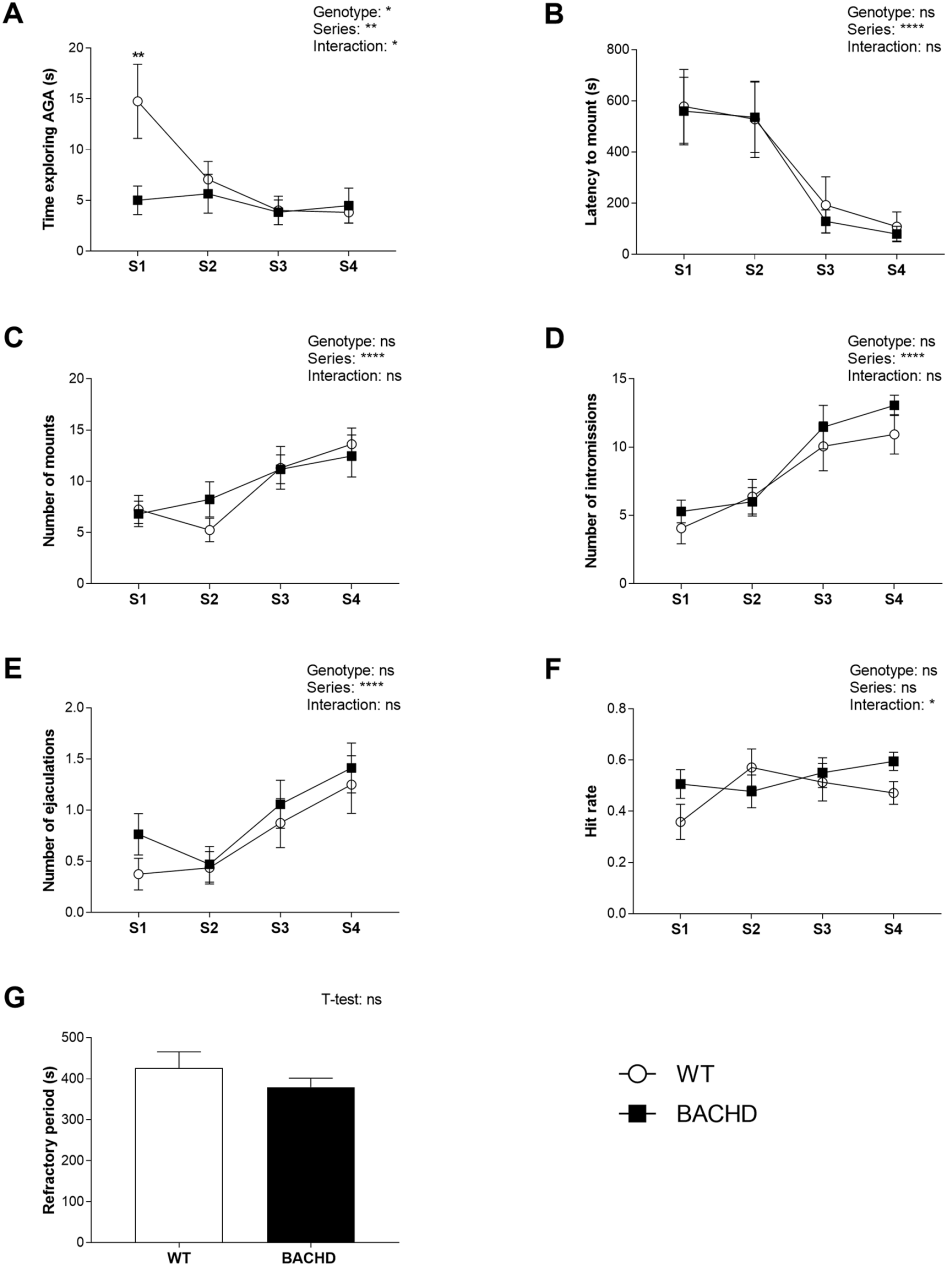
Sexual behavior at 7 months of age. The time spent exploring the AGA was lower in BACHD rats compared to WT rats in the first test series (A) while latency to mount (B), number of mounts (C), intromissions (D) and ejaculations (E) as well as hit rate (F) and post ejaculatory refractory period (G) were comparable between genotypes. Values in the graphs indicate group mean and standard error for different test series. T-test and repeated measures ANOVA results are reported above the graphs. Results from post-hoc analysis are shown in the graphs for series where genotypes differed significantly. Age differences are not displayed on graphs. * (p < 0.05) ** (p < 0.01) **** (p < 0.0001), ns (not significant). The group size (N) used in statistical analyses differed among parameters, as explained in section 2.4. For number of mounts, intromission and ejaculations as well as latency to mount N = 16 WT and 17 BACHD. For post ejaculatory refractory period N = 10 WT and 12 BACHD. For hit rate, N = 13 WT and 14 BACHD. AGA = anogenital area; S = series; WT = wild type.

BACHD rats spent significantly less time than WT littermates exploring the female AGA over all test series at 3 months (Fig 1A) and only in the first test series at 7 months of age (Fig 2A). BACHD rats at 3 months showed also shorter mount latencies than WT rats, although this effect was significant only in the first test series (Fig 1B). At seven months, WT and BACHD rats displayed comparable latencies before performing the first mount (Fig 2B). While the number of mounts was comparable between genotypes (Fig 1C), the number of intromissions (Fig 1D) and ejaculations (Fig 1E) was significantly increased in three months old BACHD rats through all test series. Accordingly, the hit rate was higher in BACHD relative to WT rats (Fig 1F). There were instead no significant genotype differences in the length of the post ejaculatory refractory period measured in test series 4 (Fig 1G). At seven months, none of these parameters differed significantly between genotypes at any time point (Fig 2C, 2D, 2E, 2F and 2G).

### Testis histological and immunohistochemical analyses

**Fig 3:**
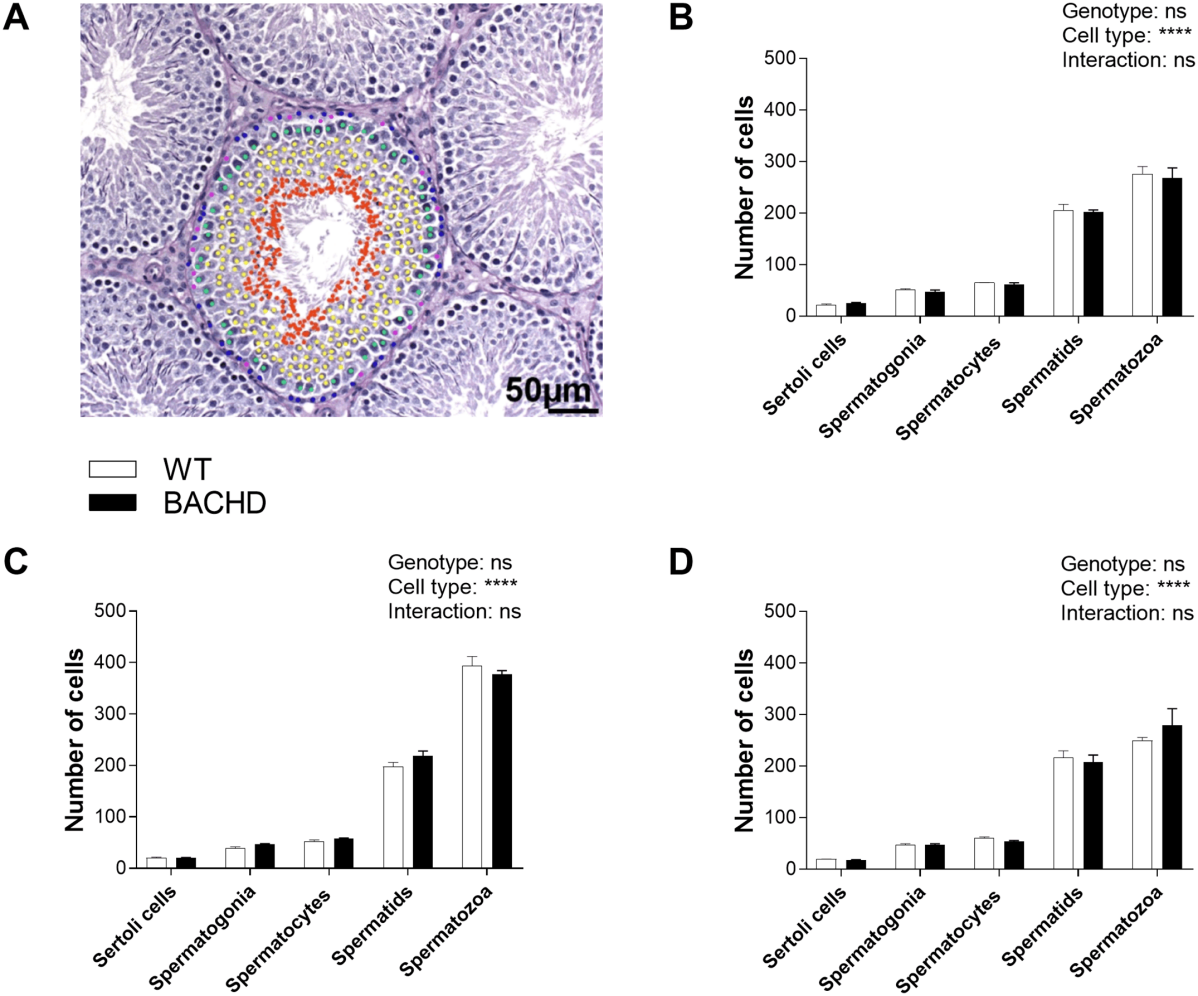
Number of cells in germinal epithelium. The representative image in (A) shows the germinal epithelium in a WT rat (200 x magnification) with Sertoli cells and germinal cells in different maturation stages (Pink: Sertoli cells; Blue: spermatogonia; Green: spermatocytes; Yellow: spermatids; Red: spermatozoa). The number of Sertoli cells and germinal cells in each stage of maturation were counted in testis of WT and BACHD animals at 1,5 (B), 7 (C) and 12 (D) months of age in 4 randomly selected tubules per animal. There were no differences in the number of cells between genotypes. Values in the graphs indicate group mean and standard error. Two-way ANOVA results are reported above the graphs. **** (p < 0.0001), ns (not significant). N = 3 WT and 3 BACHD. WT = wild type.

**Fig 4:**
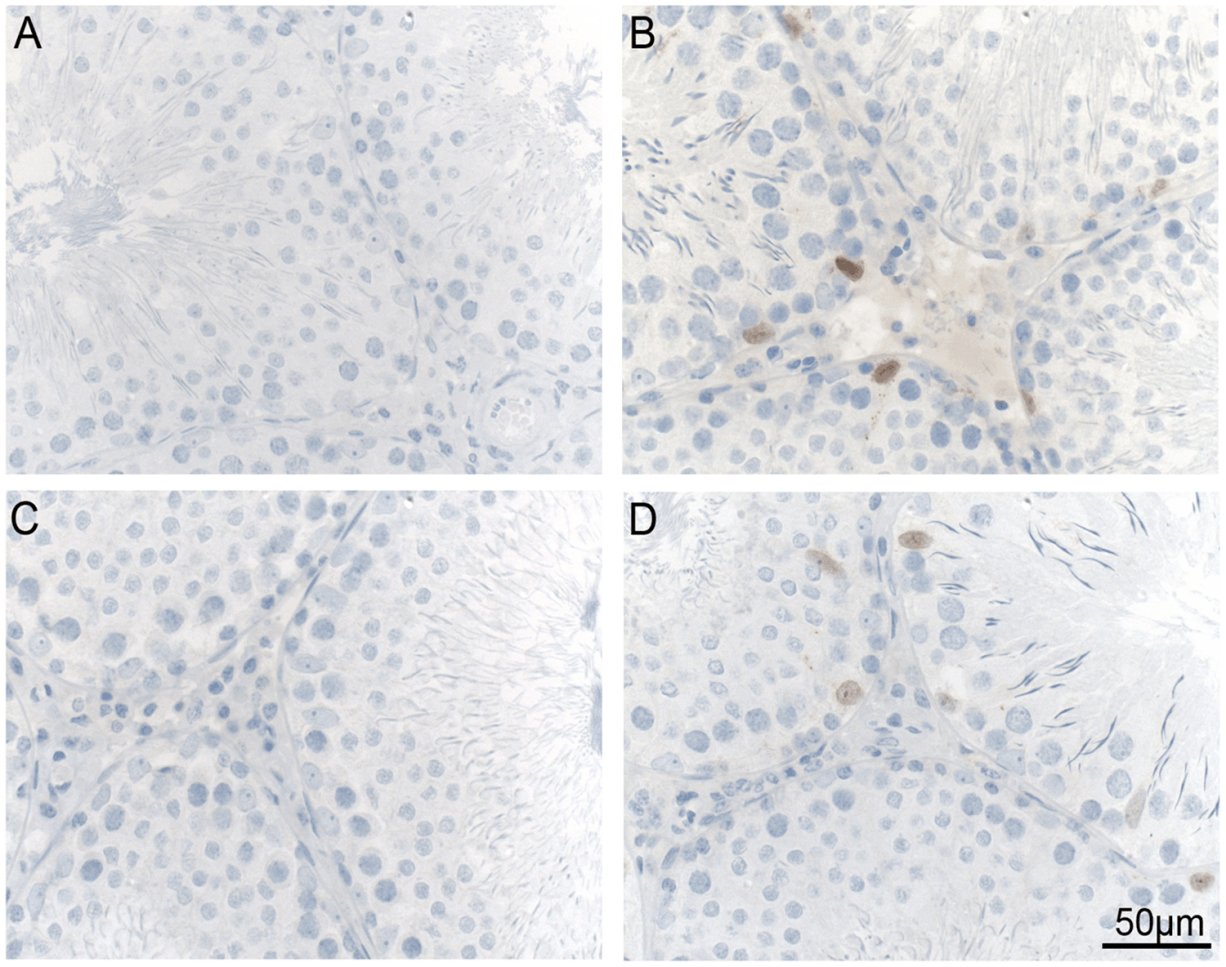
Mutant huntingtin in testis. The figure shows representative images (400 x magnification) of mutant huntingtin staining in WT and BACHD rats at 7 (A and B) and 12 (C and D) months of age. Note that WT rats are negative for mutant huntingtin (A, C) while scattered Sertoli cells in BACHD rat testis show positive nuclear staining (brown; B, D). No apparent differences are visible between 7 and 12 months of age. WT = wild type.

The number of cells counted in the germinal epithelium did not differ significantly between WT and BACHD rats for any of the maturation stages at any age (Fig 3). There were no genotype differences in tubular and germinal epithelium areas (Table 1) or in the percentage of tubules belonging to each size group (Table 2) in any of the age cohorts. Furthermore, the microscopic observation of the testicular tissue did not reveal any sign of degeneration. At both 7 (Fig 4A and 4B) and 12 (Fig 4C and 4D) months of age, mHTT was present only in BACHD rat testis and was localized at the level of Sertoli cells (Fig 4B and 4D) which were recognized based on position in the germinal epithelium and morphology (pale oval-shape nuclei and clear nucleolus). Of note, not all Sertoli cells were positive for mHTT.

**Table 1:** Germinal epithelium and tubule areas.

Age (months)	Genotype	Germinal epithelium area (μm2)	N	t-test	Tubular area (μm2)	N	t-test
**1.5**	**WT**	53884.8 (± 1194)	5	ns	67876 (± 2265.7)	5	ns
	**BACHD**	54025.7 (± 2645.2)	5		65647.2 (± 29358.3)	5	
**7**	**WT**	54026.5 (± 2357.3)	4	ns	73960.4 (± 3031.3)	4	ns
	**BACHD**	56346.1 (± 2059.2)	4		77672.4 (± 2967.6)	4	
**12**	**WT**	55915.9 (± 3519.9)	3	ns	76567.7 (± 5112.6)	3	ns
	**BACHD**	53053.2 (± 1080.6)	3		70652.8 (±1692.5)	3	

ns (not significant); WT = wild type

**Table 2:** Percentage of tubules per size group.

Age (months)	Genotype	Tubules per size group (%)				N	Two-way ANOVA
		Reduced	Small	Medium	Large		
**1.5**	**WT**	10.4 (± 2.5)	79.0 (± 2.8)	10.6 (± 4.1)	0	5	Genotype: ns Size group: **** Interaction: ns
	**BACHD**	12 (± 4.6)	81.0 (± 2.3)	6.7 (± 2.9)	0.4 (± 0.4)	5	
**7**	**WT**	2.6 (± 1.6)	75.6 (± 6)	20.9 (± 6.2)	0.9 (± 0.5)	4	Genotype: ns Size group: **** Interaction: ns
	**BACHD**	4.2 (± 2.5)	67.1 (± 8.1)	26.2 (± 6.5)	2.6 (± 1.5)	4	
**12**	**WT**	3.6 (± 2.5)	70.2 (± 7.4)	24.6 (± 9.9)	1.6 (± 0.8)	3	Genotype: ns Size group: **** Interaction: ns
	**BACHD**	3.1 (± 2.5)	83.6 (± 3.9)	13.3 (± 1.9)	0	3	

**** (p < 0.0001),

ns (not significant); WT = wild type

### Huntingtin protein expression in brain and testis

**Fig 5:**
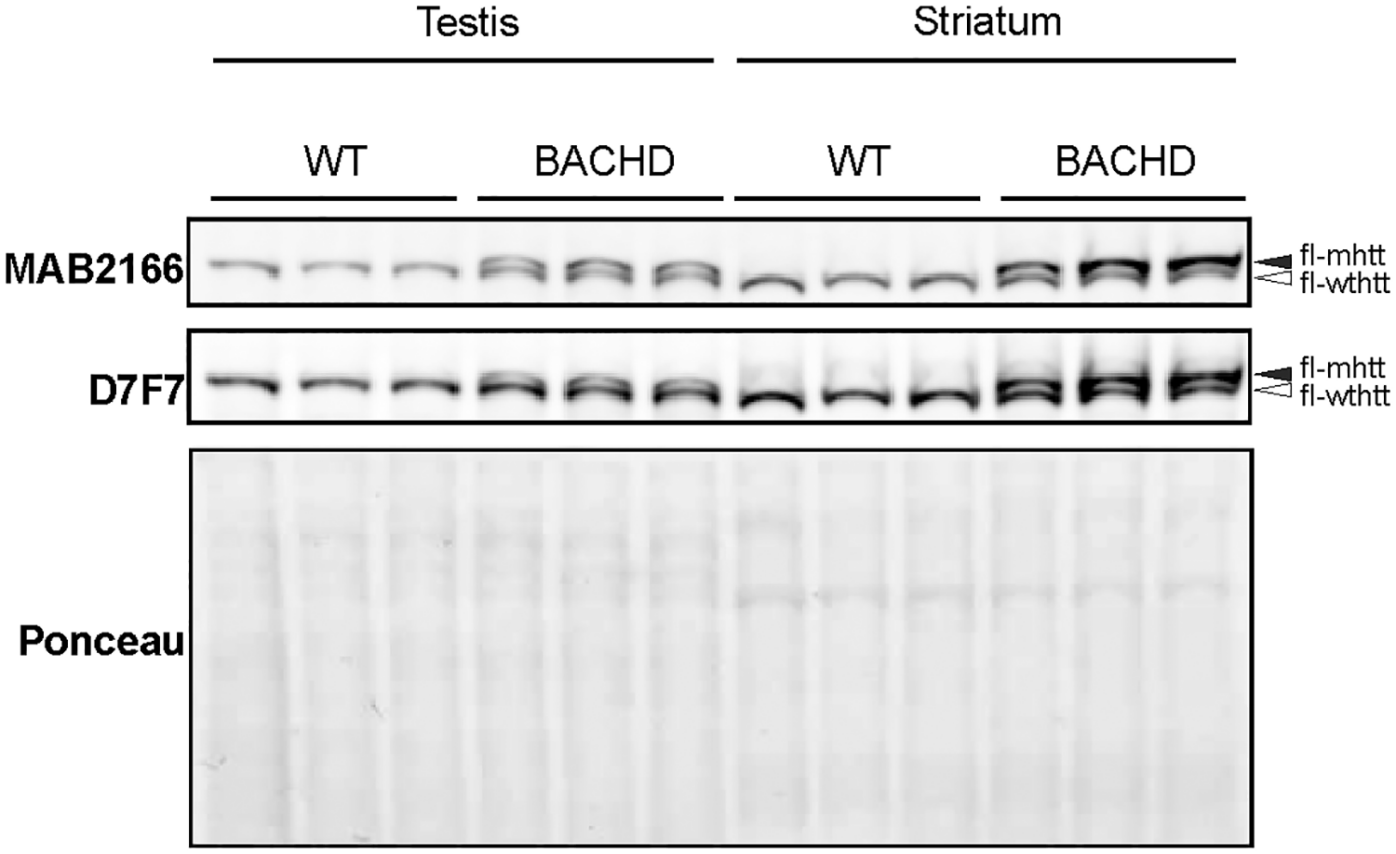
Wild type and mutant huntingtin protein expression in testis and striatum. Western blot analyses for huntingtin expression were performed in 3 months old BACHD rats and WT littermates. In order to validate the results, the blot was probed with anti HTT antibodies MAB2166 (mouse) and D7F7 (rabbit), recognizing distinct epitopes. Both antibodies detected both WT and mutant huntingtin. Ponceau red staining served to confirm equal loading of proteins. Note that WT HTT and mHTT expression levels were lower in testis than in striatum of BACHD rats. N = 3; fl-mhtt: full length mutant huntingtin; fl-wthtt: full length wild type huntingtin.

We assessed HTT expression in 3 months old BACHD rats and WT littermates using western blot analyses. Both MAB2166 and D7F7 antibodies detected WT HTT in testis and striatum of both genotypes and mHTT in testis and striatum of BACHD rats. Previous research in YAC128 mice and HD humans showed higher relative HTT protein expression levels in brain and testis compared to other organs [17,32]. Consistent with results in YAC128 mice [17], our western blot results showed abundant WT HTT protein expression in both testis and striatum (Fig 5). Differently than in YAC128 mice [17], in BACHD rats, the expression of both WT HTT and mHTT were lower in testis than in striatum (Fig 5).

## Discussion

We examined sexual behavior and testis morphology in the BACHD rat model as sexual disturbances and testis alterations have been associated with HD [7–15]. The main result in our study is that sexual behavior is abnormal in BACHD relative to WT rats at three months of age. Our findings also show presence of mHTT in Sertoli cells at both 7 and 12 months and normal testis morphology in different animal cohorts of various ages between 1.5 and 12 months.

BACHD rats at three months of age spent less time exploring the female AGA as measured in the first part of each test series and displayed shorter mount latencies in the first test series suggesting an altered precopulatory behavior in these animals. Shorter AGA exploration may also represent a decreased interest to interact with the female as shown earlier in R6/1 and R6/2 HD mouse models [33,34] or reflect general changes in social behavior for which evidence exists in BACHD rats [26]. Measuring additional precopulatory behavior parameters, e.g. ultrasonic vocalizations, in further experiments will help to understand better the precopulatory phenotype in BACHD rats. Whereas precopulatory behavior was decreased, copulatory behavior and efficiency were increased in 3 months old BACHD rats as suggested by the increased number of intromissions and ejaculations and by the enhanced hit rate respectively. These effects may indicate an increased copulatory performance in BACHD rats. Increased sexual performance has only been described in a few animal models and different studies using these models defined sexual behavior on the base of different parameters [35–37] making it difficult to compare our results to previous findings and understand the underlying mechanisms. Brain lesioning studies suggest the involvement of septal and hypothalamic areas in increasing sexual behavior [37,38]. Hypothalamic changes in HD are known in patients and animal models [39] and may affect also systems such as oxytocinergic transmission and sexual hormones in turn modulating sexual behavior and functionality [40,41]. Evidence from pharmacological studies administering methamphetamine and chlorophenylalanine in rats [35,42] further indicate a possible role of the dopaminergic and serotonergic systems in enhancing sexual behavior. Changes in dopaminergic receptors have been already shown in the BACHD rat model at the striatal level [20] and investigating the dopaminergic system in other sexually relevant areas [38] may help to understand the bases of the sexual phenotype in BACHD rats.

Increased sexual behavior in rats has been also defined as hypersexual behavior in some of the previous research [38]. The enhanced performance in our rats though is not directly translatable to hypersexuality in HD patients as hypersexuality may not be directly comparable in rats and humans. Yet, it is noteworthy that common brain areas are responsible for hypersexuality in humans and hypersexuality-like behavior in rats [38] and it is therefore possible that the sexual phenotype in BACHD rats shares some common biological bases with hypersexuality in HD subjects. One should keep in mind that sexual abnormalities in BACHD rats may also derive from or at least be influenced by other behavioral phenotypes. The decreased latency to mount the female in the first test series could be the result of an impulsivity-like phenotype which is known in BACHD rats at three months of age [25]. This is supported by a previous study showing that neurobiological changes causing impulsivity affect also male sexual behavior in rats [28]. Moreover, a shortened mount latency may be in part related to decreased anxiety levels in BACHD rats [20] and common mechanisms have even been proposed as modulators of both sexual and anxious phenotypes [43].

The lack of difference in sexual performance between genotypes at 7 months could have different explanations. One could expect that the progression of the disease at later ages results in a worsening of behavioral alterations e.g. motor impairment [20,21] that would prevent the expression of an increased sexual performance. On the other hand, unknown developmental deficits in the control of sexual behavior may play a role as well. It could be that BACHD rats go first through a phase of deficient precopulatory behavior and enhanced copulatory behavior before developing the normal sexual behavior patterns. Regrettably, it is not known how sexual disturbances evolve longitudinally in HD patients and therefore we cannot compare the changes in our model with those in humans. In order to prove the reasons at the base of the discrepancy in sexual performance at different ages in BACHD rats, it will be necessary to examine how the sexual phenotype develops in older animals and how sexual performance relates to key regulatory factors at brain and hormonal level. In this study we did not measure plasma testosterone, whose metabolites play an important role in male rat sexual behavior [44]. The absence of alterations observed in Leydig cells that are responsible for testosterone biosynthesis [45], doesn’t support the view of an impaired testosterone production in BACHD rats. Accordingly, earlier research in our lab failed to show changes in plasma testosterone levels in three months old BACHD rats (Yu-Taeger, unpublished results). For a better understanding of the sexual phenotype in the BACHD rat model, new studies will follow up the relation between sexual performance and hormonal levels by measuring both parameters in the same animals and taking into account the hormonal response to female exposure.

Our study did not aim to link sexual performance with testis morphology directly and the results do not support any relation between these two aspects in BACHD rats, although testis analyses were not examined at 3 months of age, when changes in sexual behavior where found. Testis features in our rats do not mimic the testicular pathology in HD patients and are not in line with findings in other animal models showing testicular degeneration and decreased number of germ cells [17,18]. BACHD rat testis were however, as expected, positive for mHTT when stained with EM48 antibody. The presence of mHTT in BACHD rat Sertoli cells does not seem to have affected their function in regulating spermatogenesis [46] as the number of developing cells in BACHD rats was comparable to those in WT rats. Why mHTT was detected selectively in Sertoli cells and only in a part of them, remains an interesting question, which will be considered in future research. Although EM48 stained areas in BACHD rat testis did not seem to consist of aggregates, we can not exclude the presence of small mHTT aggregates. Previous research in both YAC128 mice and HD patients failed to demonstrate mHTT aggregation in testis, but showed high levels of expression of WT and mutant HTT in brain and testis relatively to other organs [17]. Results in *Hdh* knockout mouse models suggested also an essential function of HTT in regulating spermatogenesis [47,48]. If changes in maturing cells in the germinal epithelium and testis pathology are associated with mHTT and HTT expression in testis, one would expect that the lack of changes in germinative cell number and testis morphology in BACHD rats, is paralleled by a low expression of mHTT in testis or at least a lower expression of mHTT in testis than in brain where pathological alterations are known in this model already at three months of age [20]. In line with this and in contrast with earlier results in YAC128 mice and HD humans [17], we observed lower mHTT expression levels in testis than in striatum in BACHD rats. A lower mHTT expression may have contributed to the lack of major pathological changes in BACHD rat testis and may partly explain the different testis phenotype between BACHD rats and YAC128 mice.

In conclusion, the results of this study expand the knowledge on the BACHD rat model phenotype to sexual behavior, which is altered in HD patients, but still scarcely investigated in animal models. While the intact testis morphology in BACHD rats does not resemble the testicular pathology in humans and other animal models, the presence of mHTT in Sertoli cells is an interesting finding which deserve deeper examination. The increased sexual performance in BACHD rats at young age may be in line with hypersexuality in HD patients or at least have common underlying pathways. Further research aimed to clarify the biological causes of sexual abnormalities in BACHD rats may contribute to understand the mechanisms at the base of the sexual disturbances reported in HD subjects.
